# Multiple Molecular Targets Associated with Genomic Instability in Lung Cancer

**DOI:** 10.1155/2019/9584504

**Published:** 2019-07-01

**Authors:** Giovanny Soca-Chafre, Angelica Montiel-Dávalos, Inti Alberto De La Rosa-Velázquez, Claudia Haydeé Saraí Caro-Sánchez, Adriana Peña-Nieves, Oscar Arrieta

**Affiliations:** ^1^Personalized Medicine Laboratory, Instituto Nacional de Cancerología (INCAN), Mexico; ^2^Basic Research Division, Instituto Nacional de Cancerología (INCAN), Mexico; ^3^Genomics Laboratory, Instituto Nacional de Ciencias Médicas y Nutrición, Mexico; ^4^Department of Pathology, Instituto Nacional de Cancerología (INCAN), Mexico; ^5^Palliative Care Unit, Instituto Nacional de Cancerología, México City, Mexico; ^6^Thoracic Oncology Functional Unit (UFOT), INCAN, Mexico

## Abstract

Lung cancer (LC) is the first cause of cancer-related deaths worldwide. Elucidating the pathogenesis of LC will give information on key elements of tumor initiation and development while helping to design novel targeted therapies. LC is an heterogeneous disease that has the second highest mutation rate surpassed only by melanoma, since 90% of LC occurs in tobacco smokers. However, only a small percent of smokers develops LC, indicating an inherent genomic instability. Additionally, LC in never smokers suggests other molecular mechanisms not causally linked to tobacco carcinogens. This review presents a current outlook of the connection between LC and genomic instability at the molecular and clinical level summarizing its implications for diagnosis, therapy, and prognosis. The genomic landscape of LC shows widespread alterations such as DNA methylation, point mutations, copy number variation, chromosomal translocations, and aneuploidy. Genome maintenance mechanisms including cell cycle control, DNA repair, and mitotic checkpoints open a window to translational research for finding novel diagnostic biomarkers and targeted therapies in LC.

## 1. Introduction

**Figure 1:**
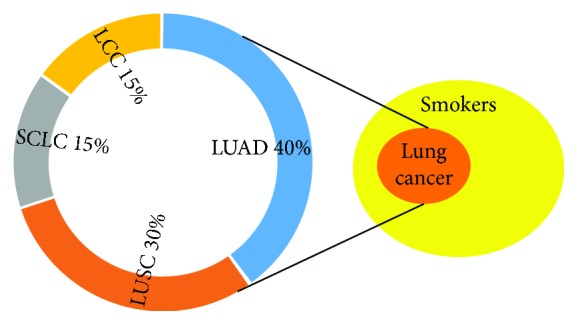
A fraction of smokers develops LC indicating subjacent germline genomic instability. LUAD: lung adenocarcinoma; LUSC: lung squamous carcinoma; LCC: large cell carcinoma; SCLC: small Cell LC.

Lung cancer (LC) remains as the most common cause of cancer-related deaths. Histologically, LC is classified into two subtypes: non-small-cell (NSCLC) and small cell (SCLC) lung cancer, 85% and 15% of LC cases, respectively. NSCLC further divides into adenocarcinoma (50%), squamous cell carcinoma (30-40%), and large cell carcinoma (10-20%) (Figure 1). Adenocarcinoma is the most frequent subtype of NSCLC independently of sex, age, or smoking history, whereas squamous cell and large cell carcinoma are strongly associated with smokers. NSCLC is often diagnosed as advanced metastatic disease, but vascular invasion is also frequent at early stages leading to recurrence and poor survival. Conventional treatments include surgery for local tumors and platinum-based chemotherapy for systemic disease. Despite current advances, NSCLC is a highly recurrent malignancy with a 5-year survival rate around 15%. Nowadays, personalized therapies are the best alternative for advanced NSCLC with known driver somatic mutations, improving clinical outcomes and quality of life for these patients. Additionally, several molecular subsets of NSCLC have distinct responses to treatment indicating that its genomic instability can be effectively harnessed for therapeutic interventions [1].

Genomic studies reveal that LC carries hundreds of somatic mutations, copy number alterations, and genome duplications. The high somatic mutation rate observed in LC results from tobacco carcinogen exposure combined with germline genomic instability. While most mutations are classified as passengers, a few driver mutations are responsible for cancer onset and progression. A better understanding of these molecular events in LC pathogenesis and evolution may allow to elucidate how tumors arise and progress, thus leading to new therapeutic options. The high genomic diversity within LC tumors is evidenced by the different subclonal populations isolated from a single biopsy. Extrinsic and intrinsic factors like tobacco smoking and DNA repair alterations contribute to frequent genomic alterations in LC. However, only a minor fraction of smokers develops cancer, highlighting the contribution of germline genetic susceptibility to lung carcinogenesis [2] (Figure 1). It is still unclear how both exogenous and endogenous processes lead to driver somatic alterations in LC, and this is the main objective of present-day genomic investigations [3]. Here, we present an overview of somatic genomic alterations in LC, the processes that contribute to its genomic instability and pathogenesis, as well as the association with clinical outcomes. We also address new therapeutic targets and specific therapies that can be developed to improve the survival and quality of life of patients with LC.

## 2. Somatic Genomic Alterations

LC exhibits a distinct genomic profile when contrasted with other types of cancer. Tobacco smoking-related LC is only second to melanoma among cancers with high somatic mutational burden [4]. The high somatic mutation rate (8-10 mutations/Mb) independent of histologic subtype in smokers, compared to less than 1 mutation/Mb in nonsmokers, supports causality of tobacco carcinogens [5]. Additionally, transversion rates (C-A) are unusually high in smoking-related LC, both in squamous cell carcinoma and in adenocarcinoma, with the highest frequency compared to other cancer types, surpassed only by melanoma rates derived from exposure to UV light, while in most cancer types, transitions are more frequent [6]. The complex genome of LC also results from endogenous mutational processes and exhibits frequent nonsilent mutations, copy number alterations (CNA), chromosomal translocations, and genome doublings [7]. There is a high genomic diversity within primary tumors in NSCLC. Spatial and temporal dynamics of tumor evolution shows that beyond tobacco smoking, high somatic mutational burden is associated with germline polymorphisms. Tumor driver mutations distribute heterogeneously and can be dominant in some clones while absent in others. Surprisingly, smoking-related mutations decay after prolonged exposure to tobacco carcinogens, and endogenous-derived mutations take over during tumor evolution [3].

The distinct genomic profile of LC defines various molecular subsets of patients and is the rationale for personalized therapies targeting driver mutations. Over the past decade, a number of tyrosine kinase inhibitors (TKIS) have been developed for *EGFR* mutations and *ALK* fusions while next-generation sequencing is revealing novel molecular targets. Data from many sequencing studies show different genomic landscapes according to histological type. Lung adenocarcinoma frequently presents mutations in receptor tyrosine kinases whereas they are rare in squamous or large cell carcinomas. This heterogeneity is further enriched by differences between mutational patterns of smokers *vs.* never smokers [5].

## 3. Mechanisms of Genomic Instability

The main etiologic factor for LC is cigarette smoking, accounting for 90% of the cases. However, around 10% of people with smoking history develops LC (Figure 1). This points out that inherent germline genomic instability may be involved in LC pathogenesis. Poor prognosis in LC relates to frequent advanced stage at the time of diagnosis, highlighting the need for early LC detection. Improved diagnostic tests could take advantage of the early events in LC pathogenesis like the molecular mechanisms of genomic instability which are known prognostic factors. The heterogeneity present in primary lung tumors is associated with genomic instability mechanisms as well as the diverse phenotypes, characteristic of LC patients. A certain degree of genomic instability correlates with poor prognosis; conversely, excessive genomic alterations lead to better prognosis affecting cancer cell replication and rendering tumors more sensitive to chemotherapy. High mutation burden results in neoantigen overexpression making tumors more immunogenic and responsive to immunotherapies. These opens therapeutic windows to exploit more cancer vulnerabilities [8].

**Figure 2:**
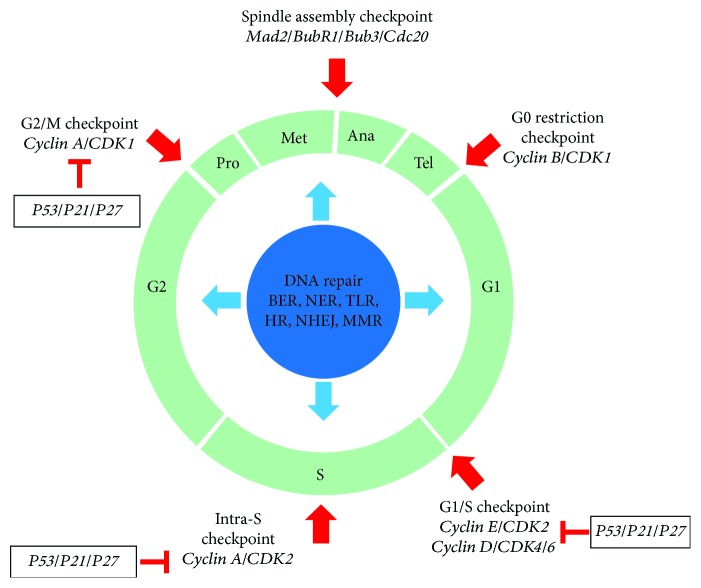
Overview of molecular checkpoints related to genomic instability in LC. BER: base excision repair; NER: nucleotide excision repair; TLS: translesion synthesis, HR: homologous recombination repair; NHEJ: nonhomologous end joining; MMR: DNA mismatch repair.

The molecular mechanisms underlying genomic instability are related to processes that preserve genetic information, namely cell cycle checkpoints, DNA repair, transcription, replication, epigenetic control, chromatin remodeling, and chromosome segregation during mitosis (Figure 2). Different types of genetic instability include chromosomal instability, microsatellite instability, and base-pair mutations. Cell cycle checkpoints are responsible for cell cycle arrest, blocking cell proliferation at different phases, and allowing the cell to repair DNA damage before division. Under irreversible DNA damage, cells develop senescence or apoptosis. Progression through different phases of the cell cycle is controlled by cyclins, cyclin-dependent kinases (*CDKs*), tumor suppressor genes, and oncogenes. These checkpoints control transitions between cell cycle phases. Defective cell cycle control is a feature of genomic instability causing chromosomal alterations and eventually malignant transformation [9]. During normal cell cycle, DNA damage occurs and is repaired by specific cellular pathways. Failure to repair DNA damage can produce mutations that accumulate and ultimately trigger carcinogenesis. Malignant diseases often present mutations affecting DNA repair genes as evidenced in more than half of NSCLC patients harboring mutations in the tumor suppressor gene *TP53* while *ATM* expression is lost in over 40% of lung adenocarcinomas [10].

## 4. Chromosomal Instability

Cancer genomes are characterized by frequent chromosomal aberrations, in the form of either point mutations, minor insertions, or deletions, as well as large chromosomal rearrangements. Chromosomal instability is considered a cancer hallmark and a driving force behind tumor heterogeneity and evolution resulting in drug resistance and reduced clinical response rates. This feature arises during cell division due to aberrant chromosome replication and segregation to daughter cells and can be either numerical or structural depending on whether whole chromosomes or segments are gained or lost. Chromosome instability results in aneuploidy, a process that can become unstable and stimulate tumor heterogeneity generating multiple cell clones with different ploidy. Extensive chromosomal instability can cause genome chaos in different cancer types experiencing high and complex genomic rearrangements. High chromosomal missegregation occurs in cancer cells (one in five cell divisions) compared to healthy cells (one in hundred divisions). The molecular mechanisms associated with chromosomal instability include defective DNA repair and replication as well as defects in kinetochore-microtubule attachment, spindle assembly, sister chromatid cohesion, and telomere dysfunction [11].

Over 60% of NSCLC cases present aneuploidy, *i.e.*, an abnormal number of chromosomes, while more than 40% of LC exhibits genome duplication. DNA content and chromosome counts show elevated chromosomal instability circulating tumor cells of NSCLC patients with *ROS1* fusions treated with crizotinib. High *ROS1* copy number and aneuploidy associate with disease progression and may be a resistance mechanism for targeted therapies [12]. In contrast, extensive chromosomal instability can confer better prognosis in LC and other cancer types and therapeutic strategies targeting this vulnerability can effectively inhibit tumor growth [13]. Aside from neoplasias, chromosomal disorders are the cause of different congenital diseases such as the Klinefelter syndrome with high risk of developing lung and breast cancer and leukemia [14]. Chromosomal aberrations are sufficient to initiate carcinogenesis as observed in knockout mice spontaneously generating LC tumors when lacking *Mad-1*/2 proteins associated with the spindle assembly checkpoint (SAC). Chromosomal instability is also responsible for developing metastasis even after oncogene withdrawal in *KRAS*-driven LC [15]. However, as mentioned above, chromosomal alterations at high scale can have protective effects reducing angiogenesis and promoting apoptosis in LC [16].

## 5. Microsatellite Instability

Deficient mismatch repair (MMR) during replication leads to multiple mutations at repetitive DNA sequence stretches, a feature known as microsatellite instability. These are short, 1-6 base pair (bp) DNA sequences ubiquitous throughout the human genome, in both exons and introns. Replication errors are frequent within microsatellites given their repetitive nature and can be repaired through MMR. However, deficient MMR proteins can produce high mutation rates in microsatellites, affecting exons relevant to cell control mechanisms, thus promoting carcinogenesis. Microsatellite alterations generally derive from germline mutations in any of the 4 MMR proteins (*MSH2*, *MSH6*, *MLH1*, *PMS2*) or through promoter hypermethylation. The frequency of microsatellite alterations varies widely among different cancer types, and some *loci* can be specific of tumor type. The Lynch syndrome is also caused by microsatellite instability, and these patients develop different malignancies at an early age including colorectal, ovarian, and stomach cancers [17].

Microsatellites are highly mutated and polymorphic and represent biomarkers in forensic studies and population genetics, also having prognostic value in different cancers. The profile and prevalence of microsatellite instability in LC is still controversial, but it is associated with initial stages of carcinogenesis [18]. Microsatellites can help to stratify LC patients for immunotherapy given their higher tumor mutational burden (TMB), neoantigen expression, and increased tumor-infiltrating lymphocytes [19]. MMR deficiency is measured through microsatellite instability and correlates with TMB levels. However, the prevalence of microsatellite instability in NSCLC in a comprehensive study of 480 lung adenocarcinomas using a mononucleotide marker panel and immunohistochemical staining of MMR proteins was only 0.8%, predominantly in smokers and early stage of diagnosis [20], while another study detected only 1 case of 341 lung adenocarcinomas (0.3%) [21].

## 6. Cell Cycle Checkpoints

Several environmental carcinogens including pollution, cigarette smoke, UV light, or internal factors such as reactive oxygen species derived from metabolism can cause DNA damage. The response to such genotoxic agents is carried out by cell cycle checkpoints that induce cell cycle arrest until DNA defects are repaired. Malfunction of cell cycle controls can result in genomic instability and the accumulation of mutations that are involved in chronic degenerative processes including aging, cerebrovascular diseases, and cancer. Cell cycle control involves different regulatory proteins such as cyclin proteins, cyclin-dependent kinases (*CDKs*), and tumor suppressors. The main regulation of transition through the four phases of cell cycle, *i.e.*, mitosis (M), gap1 (G1), DNA synthesis (S), and gap2 (G2), is based on complexes formed by cyclins and *CDKs* (Figure 2). The latter become activated and phosphorylate specific proteins including tumor suppressors that also control cell cycle progression and division. Among tumor suppressors, *p53* and *pRb* play important roles in cell cycle regulation. DNA damage activates *p53* which promotes *p21* transcription inhibiting CDKs that in turn phosphorylate *pRb* and block DNA synthesis. *TP53* also triggers apoptosis in the event of irreversible DNA damage [22].

In LC, high levels of *CDK4*, a Ser/Thr protein that regulates the G1 phase, have been detected in primary and metastatic tumors. *CCNY*, a cyclin regulating cell division cycles, is amplified in metastatic tumors and correlates with tumor progression [23]. *CDKN3*, a spindle checkpoint phosphatase, is overexpressed and related to worse prognosis in LC [24]. More than half of LC patients with *EGFR* mutations also carry *CDK* aberrations including *CDK4/6* or *CDKN2A/B* [25]. *TP53* tumor suppressor is highly mutated in human cancers with a prevalence over 50%. Both cell lines and LC tumors have frequent *TP53* mutations that override cell cycle control and in concert with *KRAS* mutations lead to tumorigenesis and metastasis [26]. *TP53* mutations correlate with resistance to cisplatin-based chemotherapy in NSCLC by activating *Nrf2*, a transcription factor encoding detoxification enzymes with negative impact on overall survival [27]. Therapeutic approaches targeting cell cycle control include inhibition of *CDKs 4/6* with palbociclib, *CDKs 1/2* with dinaciclib, cyclin *D1/CDK4* with simvastatin, and multiple *CDKs* with the pan-*CDK* inhibitor roniciclib, and AZD1775 inhibits *Wee1*, a tyrosine kinase that regulates G2/M and blocks mitotic progress in the presence of DNA damage (Table 1).

## 7. DNA Repair Mechanisms

Genomic instability also derives from errors in DNA repair mechanisms including base excision repair (BER), nucleotide excision repair (NER), translesion synthesis (TLS), homologous recombination repair (HR), nonhomologous end joining (NHEJ), and DNA mismatch repair (MMR) (Figure 2). The genomic DNA in a single cell receives over 10 000 lesions daily including base changes and single-strand breaks (SSB). BER is the major mechanism correcting these alterations as well as alkylation and other oxidative damages. The NER pathway can repair different DNA lesions at the nucleotide level, such as DNA crosslinks, pyrimidine dimers, or bulky adducts caused by UV light or tobacco smoke carcinogens. TLS is a repair mechanism by which specialized DNA polymerases can bypass DNA damage and perform efficient DNA replication in the presence of thymine dimers, double-strand DNA, and apurinic/apyrimidinic sites [28]. SSB can be effectively removed by BER or NER mechanisms, but double-strand breaks (DSB) are more lethal and can only be repaired by HDR or NHEJ pathways. Additionally, MMR is a mechanism that repairs mismatched bases resulting from DNA replication, recombination, or physical lesions [29].

Germline polymorphisms in *XRCC1* from the BER mechanism can downregulate the promoter and reduce gene expression conferring higher risk of NSCLC whereas CpG island hypermethylation of this gene positively correlates with lung carcinogenesis [30]. Germline polymorphisms in several mediators of the NER pathway including *XPA*, *RPA1*, *POLD1*, and *POLD3* correlate with clinical response to platinum-based chemotherapy in NSCLC [31]. Additionally, Pol *η* level in NSCLC cells, a specialized polymerase participating in TLS, has a negative impact on the response to cisplatin treatment [32]. *DEK* is an ubiquitous chromatin-bound protein playing a key role in HDR-mediated repair of DSBs. *DEK* loss of function in different malignancies including LC makes cells more sensitive to chemotherapy, while *DEK* overexpression results in shorter disease-free and overall survival [33]. Likewise, one core factor of NHEJ mechanism, *DNA-PKcs*, is overexpressed and mediates radioresistance in LC [34]. In contrast, MMR proteins *MSH2* and *MLH1* are reduced in over half of lung adenocarcinomas and associate with poor prognosis [35]. Targeted therapies for DNA repair elements comprise several specific inhibitors for the BER pathway, such as olaparib that blocks *PARP* activity, M6620 that targets *ATR*, and different indenone derivatives that block *AlkBH3*, while NSC16168 inhibits *ERCC1-XPF* from the NER mechanism and NU7026 impairs *DNA-PKcs* activity in NHEJ (Table 1).

## 8. The Mitotic Checkpoint

The G1 and G2 checkpoints arrest cells to allow DNA repair whereas the spindle assembly checkpoint (SAC) is an evolutionarily conserved surveillance mechanism that drives correct chromosomal segregation to daughter cells. SAC arrests cells in metaphase until the mitotic spindle is assembled and attached to the sister chromosomes during chromosome biorientation. Regulatory proteins involved in this process include members of the budding uninhibited by benzimidazole (*Bud*) and mitotic arrest-deficient (*Mad*) families and other components of the spindle checkpoint that prevent aneuploidy (Figure 2). The mitotic checkpoint pathway is activated by defects in kinetochore-microtubule binding and forms the mitotic checkpoint complex (MCC) that inhibits the anaphase-promoting complex (APC/C), an ubiquitin ligase that stimulates the transition from metaphase to anaphase by targeting *securin* and *cyclin B* for ubiquitination and degradation; thereby, MCC prevents chromosome segregation. MCC includes different proteins like *Mad1*, *Mad2*, *Mad3* (*BubR1*), *Bub1*, *Bub3*, *Cdc20*, and *MPS1/Mph1*. The *Mad2* protein interacts with *Mad1* that is attached to the kinetochore; *Mad2* then undergoes conformational changes and binds *Mad3-Bub3-Cdc20* to assemble MCC. When chromosomes are properly attached to the mitotic spindle, MCC no longer blocks APC/C starting the anaphase [36].

Somatic mutations in the multidomain protein kinases *BUB1* and *BUBR1*, components of MCC as well as functional deregulation, have been detected in LC patients and cell lines [22]. Additionally, germline polymorphic variants of mitotic checkpoint-related genes *BUB3*, *AURKB*, and *PTTG1* negatively influence overall survival of NSCLC patients [37]. Germline polymorphisms in other SAC components, *MAD1L1* and *MAD2L1*, affect protein structure, altering the spindle checkpoint activity, thereby promoting aneuploidy, tumorigenesis, and increased LC risk [38]. Both proteins are overexpressed in lung adenocarcinoma, have negative impact on relapse-free and overall survival, and represent potential targets for diagnosis and treatment [39]. S-phase kinase-associated protein 2 (*Skp2*) is overexpressed in LC, and blocking its activity enhances response to paclitaxel and reduces *Mad2* levels and *pRB* phosphorylation while increasing *p27* protein, being a potential target for LC therapies [40]. At this moment, there are few available inhibitors for SAC components; these include CFI402257 and TC Mps1 12 against monopolar spindle 1 (*MPS1*) or *TTK* and the BI2536 and LY3295668 inhibitors of *PLK1* and *AurA*, respectively, while *CDC20* is highly expressed and poor prognostic factor therefore constitutes an attractive biomarker and therapeutic target in LC (Table 1).

## 9. Conclusions

The major molecular mechanism underlying genomic instability in LC is defective cell cycle control, involving cyclins, cyclin-dependent kinases, tumor suppressors, and oncogenes. Also, aberrant DNA repair mechanisms including BER, NER, TLS, HR, NHEJ, and MMR and malfunction of components in the spindle assembly checkpoint such as members of the *Bud* and *Mad* families contribute to genomic instability. This represents opportunities for the development of novel biomarkers and targeted therapies since many of these proteins correlate with cancer cell growth, survival, tumorigenesis, and metastasis in preclinical models both *in vitro* and *in vivo* while they are overexpressed and/or have prognostic value for LC patients.

## Figures and Tables

**Table 1:** Recent molecular therapies and biomarkers targeting elements of genomic instability in LC.

	Target	Therapy	Reference	Rationale
Cell cycle	*Wee1*	AZD1775	[41]	Study of a selective inhibitor of the tyrosine kinase *Wee1* that impairs *CDK1/2* activity inducing cell cycle arrest at G2 and intra-S checkpoints.
	*CDK4/6*	Palbociclib	[42]	Blocking cyclin-dependent kinases *CDK4* and *CDK6* represses the G1/S transition in the cell cycle inhibiting cancer cell proliferation.
	*CDK1/2*	Dinaciclib	[43]	Dinaciclib inhibits *CDK1* and *CDK2* inducing anaphase catastrophe, apoptosis, and cell death and represents a potential treatment for LC.
	*Pan-CDKs*	Roniciclib	[44]	Cancer cells overexpress cyclins and *CDKs*; therefore, blocking multiple *CDKs* induces cell cycle arrest and apoptosis and reduces tumorigenesis.
	*Cyclin D1/CDK4*	Simvastatin	[45]	Simvastatin downregulates protein levels of *cyclin D1* and *CDK4* while upregulating expression of *p16* and *p27* that inhibit *CDK* activity.

DNA repair	*PARP*	Olaparib	[46]	A poly(ADP-ribose) polymerase (*PARP*) inhibitor that blocks DNA repair and in combination with other agents can induce synthetic lethality in cancer cells.
	*ATR*	M6620	[47]	An ataxia telangiectasia and rad3-related (*ATR*) kinase inhibitor that blocks DNA repair after damage induced by the chemotherapeutic agent topotecan.
	*AlkBH3*	Indenone derivatives	[48]	AlkB homologue-3 (*AlkBH3*) participates in direct reversal of DNA damage. Inhibiting this dealkylation enzyme prevents carcinogenesis and metastasis.
	*ERCC1-XPF*	NSC16168	[49]	*ERCC1-XPF* participates in the NER mechanism removing DNA adducts caused by cisplatin; targeting this enzyme can sensitize cells to chemotherapy.
	*DNA-PKcs*	NU7026	[50]	The catalytic subunit of DNA-dependent protein kinase (*DNA-PKcs*) is fundamental for DNA double-strand break (DSB) repair and radiosensitivity.

Mitotic control	*PLK1*	BI2536	[51]	*PLK1* binds to *Bub1* and contributes to the regulation of proper chromosome segregation by stabilizing the spindle assembly checkpoint (SAC) complex.
	*TTK*	CFI402257	[52]	Monopolar spindle 1 (*MPS1*) or *TTK* regulates SAC activity being fundamental for genome maintenance during mitosis; therefore, it is an attractive target.
	*AurA*	LY3295668	[53]	Retinoblastoma (*RB1*) protein regulates G1/S transition, together with the mitotic checkpoint *AurA* that creates synthetic lethality in refractory LC.
	*Mps1*	TC Mps1 12	[54]	This inhibitor of a SAC element leads to accumulation of chromosomal alterations by missegregation, *i.e*., aneuploidy and cytotoxicity in A549 cells.
	*CDC20*	Biomarker	[55]	*CDC20*, part of the MCC, is overexpressed and confers poor prognosis in LC exacerbating pleural invasion and reducing 5-year overall survival.
